# Automated quantitative assay of fibrosis characteristics in tuberculosis granulomas

**DOI:** 10.3389/fmicb.2023.1301141

**Published:** 2024-01-03

**Authors:** Li Song, Ding Zhang, Hankun Wang, Xuan Xia, Weifeng Huang, Jacqueline Gonzales, Laura E. Via, Decheng Wang

**Affiliations:** ^1^The First College of Clinical Medical Science, China Three Gorges University, Yichang, China; ^2^Yichang Central People’s Hospital, Yichang, China; ^3^Hubei Key Laboratory of Tumor Microenvironment and Immunotherapy, College of Basic Medical Sciences, China Three Gorges University, Yichang, China; ^4^Institute of Infection and Inflammation, China Three Gorges University, Yichang, China; ^5^Tuberculosis Research Section, Laboratory of Clinical Infectious Diseases, Division of Intramural Research, National Institute of Allergy and Infectious Diseases, National Institutes of Health, Bethesda, MD, United States; ^6^Institute of Infectious Disease and Molecular Medicine, University of Cape Town, Cape Town, South Africa

**Keywords:** fibrosis, tuberculosis, granuloma, *Mycobacterium*, second harmonic generation (SHG)/two-photon excited fluorescence (TPEF)

## Abstract

**Introduction:**

Granulomas, the pathological hallmark of *Mycobacterium tuberculosis* (*Mtb*) infection, are formed by different cell populations. Across various stages of tuberculosis conditions, most granulomas are classical caseous granulomas. They are composed of a necrotic center surrounded by multilayers of histocytes, with the outermost layer encircled by fibrosis. Although fibrosis characterizes the architecture of granulomas, little is known about the detailed parameters of fibrosis during this process.

**Methods:**

In this study, samples were collected from patients with tuberculosis (spanning 16 organ types), and *Mtb*-infected marmosets and fibrotic collagen were characterized by second harmonic generation (SHG)/two-photon excited fluorescence (TPEF) microscopy using a stain-free, fully automated analysis program.

**Results:**

Histopathological examination revealed that most granulomas share common features, including necrosis, solitary and compact structure, and especially the presence of multinuclear giant cells. Masson’s trichrome staining showed that different granuloma types have varying degrees of fibrosis. SHG imaging uncovered a higher proportion (4%~13%) of aggregated collagens than of disseminated type collagens (2%~5%) in granulomas from matched tissues. Furthermore, most of the aggregated collagen presented as short and thick clusters (200~620 µm), unlike the long and thick (200~300 µm) disseminated collagens within the matched tissues. Matrix metalloproteinase-9, which is involved in fibrosis and granuloma formation, was strongly expressed in the granulomas in different tissues.

**Discussion:**

Our data illustrated that different tuberculosis granulomas have some degree of fibrosis in which collagen strings are short and thick. Moreover, this study revealed that the SHG imaging program could contribute to uncovering the fibrosis characteristics of tuberculosis granulomas.

## 1 Introduction

Granulomas, organized structures composed of various cells, are considered to be the hallmark lesion of tuberculosis (Adams, 1976; Barry et al., 2009; Silva Miranda et al., 2012; Shi et al., 2016). As physiologically isolated regions, granulomas were originally considered necessary for containing mycobacterial dissemination and infection (Adams, 1976); however, understanding of the role of granulomas in mycobacterial infections has changed in light of accumulating reports with new views (Davis and Ramakrishnan, 2009; Russell et al., 2009; Gil et al., 2010; Ehlers and Schaible, 2012; Ramakrishnan, 2012; Cronan, 2022). Data from a *Mycobacterium marinum* (*Mm*)–larval zebrafish model system provided hints that granulomas may provide the mycobacteria with a nutrient microenvironment that “walls off” the bacteria from the host immune response, rather than simply containing them (Russell, 2007; Barry et al., 2009; Davis and Ramakrishnan, 2009; Ramakrishnan, 2012). To date, the full role of granulomas in the mycobacterial community remains enigmatic.

Three granuloma types can be found among humans and non-human primates (NHPs): classic caseous granulomas, fibrotic granulomas, and non-necrotizing granulomas (Barry et al., 2009). Conventional caseous granulomas, which have been extensively reported in active disease and latent conditions after mycobacterial infection, display a three-layered architecture: a necrotic center, viable cell areas, and an outer fibrotic rim (Adams, 1976; Russell, 2007; Barry et al., 2009; Silva Miranda et al., 2012). Because it is difficult to obtain biopsy samples from human lungs, animal models have been improved over the years to more closely duplicate the pathological progressions in humans, commonly applied in granuloma research. Mice are a practical animal model for the study of granuloma kinetics following infection with various mycobacterium species, even *Mm* (Carlsson et al., 2010). Importantly, although mice do not reproduce human tuberculosis pathology, especially the organized granulomas, mouse models have still been the most practical and widely used animal model for tuberculosis-related research, especially transgenic mouse models of chronic granulomatous disease that have normal granuloma formation and cytokine responses against *Mycobacterium avium* and *Schistosoma mansoni* eggs (North and Izzo, 1993; Iuvone et al., 1994; Kunkel et al., 1996; Segal et al., 1999; Jakubzick et al., 2002, 2004). By using an *Mm*–zebrafish infection system, Swaim et al. documented that zebrafish granulomas also undergo caseous necrosis, similar to human tuberculous granulomas, in which RD-defective *Mm*-induced granulomas are likely to be solitary, non-necrotizing, and loosely associated, unlike those in wildtype *Mm*-infected zebrafish (Swaim et al., 2006). To date, various animal models have been established and used to explore the mycobacterium–host interaction and the determinants of granuloma formation and development; these models have generated valuable information and improved our understanding of the host–pathogen relationship.

Fibrosis, a critical response to injury and/or inflammatory processes, is a pathophysiological consequence of chronic wound healing in which excess extracellular matrix is deposited (Diegelmann and Evans, 2004; Strieter, 2008). In an NHP model of *Mycobacterium tuberculosis* (*Mtb*) infection, granulomatous lesions were formed and surrounded by fibrillar collagens (DiFazio et al., 2016). Collagen, the most abundant mammalian protein, is not only the main component of connective tissues but it also provides the tensile strength of organs. This protein is extremely resistant to degradation but can be cleaved by matrix metalloproteinase (MMP). Interestingly, MMP-1 can degrade fibrillar collagens during *Mtb* infection, resulting in significant levels of collagen breakdown and alveolar destruction in lung granulomas (Elkington et al., 2011). MMP-9, which is homologous to MMP-1, can promote both the recruitment of macrophages to form nascent granulomas and bacterial growth (Volkman et al., 2010). Therefore, fibrosis and its associated factors play a crucial role in the immunopathology of granuloma formation.

There is an increasing need for the accurate assessment of fibrosis parameters in tuberculosis granulomas, such as the collagen content, geometric and textural features of collagen fibers, number of cross-linked collagen fibers, and physical appearance (thick or thin and short or long collagens). Unfortunately, there were few publications describing the fibrosis of tuberculosis granulomas in animal models by conventional Masson’s trichrome staining (Swaim et al., 2006), until Warsinske et al. constructed a hybrid multi-scale model of fibrotic granuloma formation in 2017 and discovered the underlying mechanism that promoted fibrosis in *Mtb*-infected lungs (Warsinske et al., 2017). By combining the three-dimensional dynamics of molecular, cellular, and tissue scale data, Warsinske et al. described two types of fibrosis during granuloma formation: 1) peripherally fibrotic granuloma, with a cuff of collagen surrounding the granuloma, and 2) centrally fibrotic granuloma, with collagen throughout the granuloma. These findings inspired research to better understand the mechanisms of fibrosis dynamics and their clinical significance in granuloma formation following *Mtb* infection.

Stemming from the recent telecommunication boost, developments in highly sensitive optical lasers have made nonlinear optical microscopies, such as multiphoton excited fluorescence and multiharmonic generation, an affordable option for tissue imaging. Over the past decades, a new type of second harmonic generation/two-photon excited fluorescence (SHG/TPEF) microscopy, termed Genesis program, was initiated and developed to observe the morphological features of fibrosis-associated parameters in hepatitis and inflammation without staining (Tai et al., 2009; Xu et al., 2014; Wang et al., 2015, 2017; Liu et al., 2017). Second harmonic generation (SHG) microscopy is a novel tissue imaging system based on non-linear optical microscopy which enables observation of endogenous tissue signals such as Two-Photon Excitation Fluorescence (TPEF) and Second Harmonic Generation (SHG) in unstained tissue samples. TPEF provides visualization of the background tissue architecture while the SHG signal provides accurate identification of fibrillar collagen. Computerized algorithms based on SHG pattern recognition and logic modeling allow automated determination of the severity of fibrosis with the added advantage of objectivity, reproducibility and fast turnaround time. SHG exhibits intrinsic advantages over conventional staining and image capturing for collagen; as it is a nonlinear optical process that does not require staining, the image quality is more consistent than that of images obtained from stained slides, and this reproducibility is particularly useful for pathological assessment in multi-center clinical studies. SHG can be used for the quantitative measurement of collagen in various organs and tissues as an indication of fibrosis initiation and development during different conditions, including inflammation, infection, and cancer. Recently, the feasibility and effectiveness of using SHG microscopy for monitoring fibrosis in the liver has been established and demonstrated, particularly in patients with chronic hepatitis (Cox et al., 2003; Gorrell et al., 2003; Sun et al., 2008; Xu et al., 2014). Xu et al. demonstrated that SHG can improve fibrosis scoring accuracy in a hepatitis B virus-related animal model as well as in patients with hepatitis B; this allows for reproducible and reliable analyses of the efficacies of anti-fibrotic therapies in clinical research (Xu et al., 2014). Liu et al. employed an SHG evaluation system to assess the liver fibrosis induced by thioacetamide, carbon tetrachloride, and bile duct ligation in rodent models and found that this system offers a reliable approach yielding impressive data for liver fibrosis studies that may be applicable for research of future anti-fibrotic drugs (Liu et al., 2017). Wang et al. used an SHG approach to assess hepatic fibrosis in nonalcoholic fatty liver disease (NAFLD) and demonstrated that SHG provides an accurate and reproducible method for evaluating fibrosis in NAFLD along a quantitative and continuous scale. However, although the application of SHG in liver fibrosis research is now common, the feasibility and effectiveness of applying the SHG system to the study of fibrosis of tuberculosis granulomas remain unknown.

In the present study, a set of 424 tissue samples from patients with tuberculosis, spanning 16 organ types, was collected and used to dissect the characteristics of granulomas from different organs. Masson’s trichrome staining was performed to detect the distribution of collagen fibers in granulomatous lesions. Finally, an SHG automated system was applied in combination with SHG-associated parameters and mycobacterium-infected animal models, to evaluate the detailed characteristics of the collagen surrounding granulomas within granulomatous lesions. Our results may provide feasible approaches for improving the accuracy for quantitative analysis of the fibrotic setting surrounding granulomas during tuberculosis development, thus allowing for potentially reproducible and reliable research on anti-fibrotic therapies for tuberculosis.

## 2 Materials and methods

### 2.1 Sample collection

**Figure 1:**
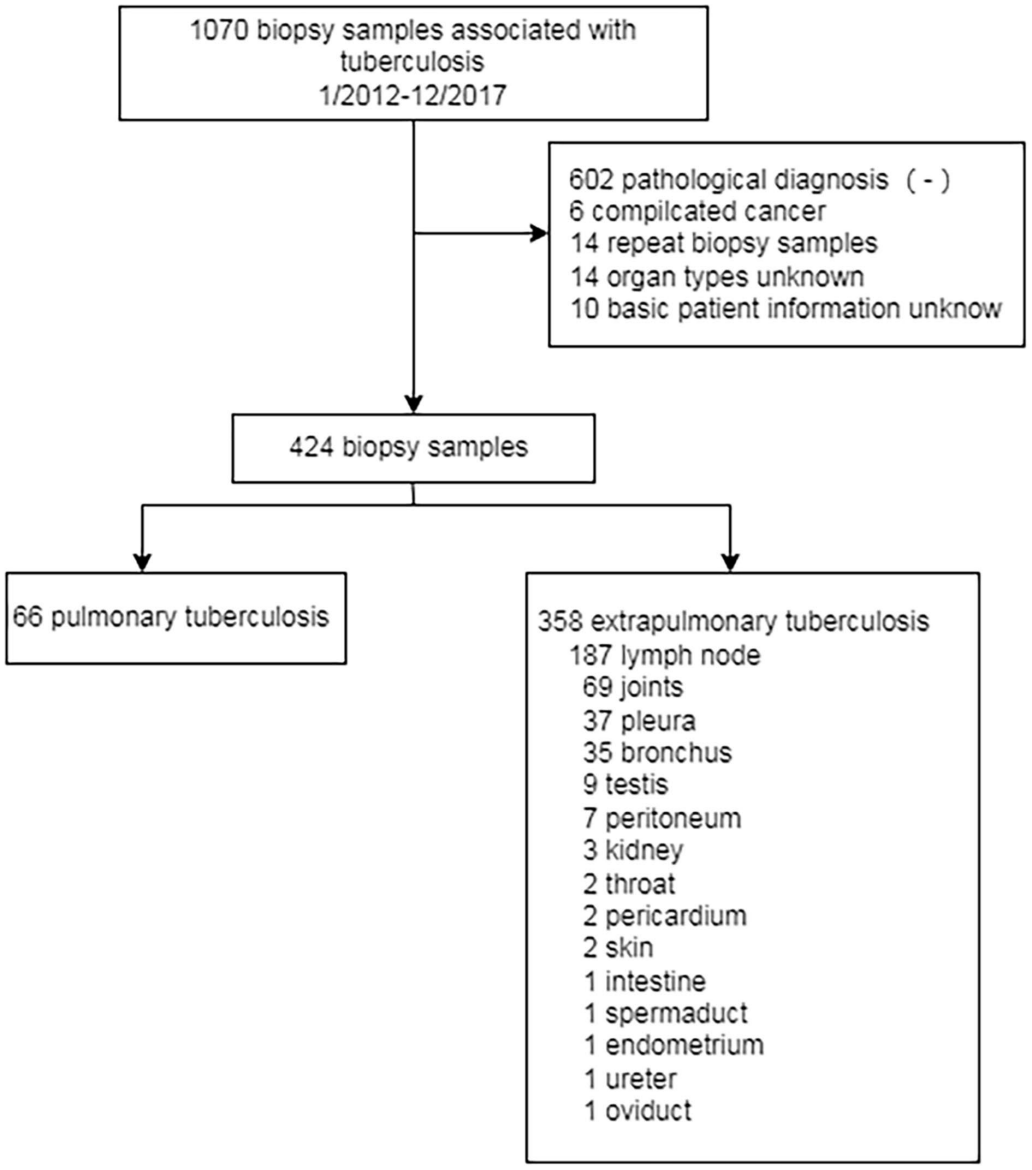
The flow chart of the designed study. 1,070 biospy have been collected from patient with clinical tuberculosis from 2012 to 2017. Only 424 samples have been chosen in this study. 66 samples belong to pulmonary tuberculosis while 358 sample belong to extrapulmonary tuberculosis.

This study collected 1,070 samples from patients diagnosed with tuberculosis who came from The First People’s Hospital of Yichang during the period from January 2012 to December 2017. After selection according to inclusion criteria and exclusion criteria, 424 samples with contact information from patients were chosen in this study (Figure 1). Inclusion criteria are as follows: patients who consented and were clinically stable for biopsies to be performed, and at least one tissue could be provided. This clinical patient must be diagnosed according to *WHO Treatment of tuberculosis: guidelines-4th* edition. WHO/HTM/TB/2009.420 (https://apps.who.int./iris/bitstream/handle/10665/44165/9789241547833_eng.pdf). Exclusion criteria are as follows: subjects had been negative according to pathological biopsy; comorbidity with carcinoma; repeated biopsy; unknown organ typing; basic information of patients is missing.

Clinical signs and pathological characteristics were obtained from the patient’s medical records (Table 1). In total, samples spanning 16 types of tissues were collected from 424 cases (Table 2); 80 of these samples were selected randomly for continuous study according to tissue classification (Table 2).

**Table 1:** Demographical information of clinical TB patients.

Basic information	Classification	Amount (percentage, %)
Gender	Male	201(47.4%)
	Female	223(52.6%)
Age	<20	33(7.8%)
	20–39	161(38.0%)
	40–59	146(34.4%)
	60–79	78(18.4%)
	≥80	6(1.4%)
History of previous TB	Yes	24(5.6%)
	No	95(22.4%)
	Unkonwn	305(72%)
TB antibody test	Positive	4(1%)
	Negative	41(9.7%)
	Uncheck	68(16%)
	Unkonwn	311(73.3%)
TB protein chip	Positive	3(0.7%)
	Negative	13(3.1%)
	Uncheck	96(22.6%)
	Unkonwn	312(73.6%)
Interferon-γ release assays of A.TB test	Positive	3(0.7%)
	Negative	7(1.7%)
	Uncheck	101(23.8%)
	Unkonwn	313(73.8%)
Sputum smear	Positive	5(1.2%)
	Negative	32(7.5%)
	Uncheck	81(19.1%)
	Unkonwn	306(72.2%)
PPD test	Positive	6(1.4%)
	Negative	0(0)
	Uncheck	104(24.5%)
	Unkonwn	314(74.1%)

TB, tuberculosis.

**Table 2:** Demographical characterization of clinical TB patients.

Tissue category	Sample size (*n*, %)
Lymph node	187(44.1%)
Joints	69 (16.3%)
Lung	66 (15.6%)
Pleura	37 (8.7%)
Bronchus	35 (8.3%)
Testis	9 (2.1%)
Peritoneum	7 (1.7%)
Kidney	3 (0.7%)
Throat	2 (0.5%)
Pericardium	2 (0.5%)
Skin	2 (0.5%)
Intestine	1 (0.2%)
Spermaduct	1 (0.2%)
Endometrium	1 (0.2%)
Ureter	1 (0.2%)
Oviduct	1 (0.2%)

The formalin-fixed, paraffin-embedded biopsy samples were acquired from the First People’s Hospital of Yichang. This study was approved by the Ethical Review Committees of the First People’s Hospital of Yichang and was conducted in accordance with the local legislation and institutional requirements. The paraffin-embedded lung tissues of *Mtb* aerosol-infected marmosets in this study were acquired from the Tuberculosis Research Section, National Institute of Allergy and Infectious Diseases (Via et al., 2015). The original animal experiment was approved by the NIAID Animal Care and Use Committee approved the experiments described herein under protocol LCID-9 (permit issued to NIH as A-4149-01) (Via et al., 2015). The study was conducted in accordance with the local legislation and institutional requirements. *Schistosoma japonicum*-infected specific pathogen-free C57BL/6 mice were established and their livers were sampled for subsequent study as previously described (Wan et al., 2017).

### 2.2 Masson’s trichrome staining

Masson’s trichrome staining was performed at the Department of Pathology, Yichang Central People’s Hospital, as follows. First, 5-μm sections were prepared, deparaffinized, and hydrated in distilled water. Bouin’s fixative was then used as a mordant at 56°C for 1 h. The formalin-fixed, paraffin-embedded sections were cooled and washed in running water until the yellow coloring disappeared. The samples were stained in Weigert’s hematoxylin stain for 10 min, thoroughly washed in tap water for 10 min, stained again in an acid fuchsin solution for 15 min, and rinsed in distilled water for 3 min. After rinsing, the slides were treated with phosphomolybdic acid solution for 10 min and then rinsed in distilled water for 10 min. Finally, slides were stained with a light-green solution for 2 min and rinsed in distilled water. After thorough dehydration using alcohol, the slides were mounted, and coverslips were placed onto them. The intensity of fibrosis-positive staining in human and animal tissues was quantified in 25 microscopy fields of view (40× magnification), and the means were calculated. Images of all sections were unbiasedly captured under an Olympus BX63 microscope by a single investigator. The results are expressed as fibrosis area density (area of positives/area of whole field).

### 2.3 SHG image acquisition system and quantification analysis

Images of unstained sections of tissue samples were acquired automatically by using the Genesis system (Hangzhou HistoIndex Ltd., Hangzhou, China) in which SHG microscopy was applied for visualizing collagen, and other cell structures were visualized with TPEF microscopy. The images were acquired at a fixed magnification with 512 × 512-pixel resolution, and each image had a dimension of 200 μm^2^ × 200 μm^2^. Ten five-by-five images were acquired for each sample to image most of each sample area, with a final image size of 10 mm^2^. Details of the optical parameters and settings were optimized based on established protocols (Tai et al., 2009; Xu et al., 2014; Wang et al., 2015; Liu et al., 2017).

The total content of collagen and previously described collagen features, including specific collagen strings and collagen connectivity-related measurements, were examined for correlations with the granuloma fibrosis scores. Nineteen morphological features were used in this study (detail seen in Supplementary Table 1). These variable parameters were divided into three groups, as follows:

Group 1: Collagen percentage of the total area (SHG), the aggregated collagen percentage of the total area (AGG), and the distributed collagen percentage of the total area (DIS). The total collagen level was detected by SHG and segmented from the raw images. The aggregated and distributed collagens were differentiated from one another with a low-pass filter.

Group 2: Collagen string number (NoStr), short collagen string number (NoShortStr), long collagen string number (NoLongStr), thin collagen string number (NoThinStr), thick collagen string number (NoThickStr), total string area (StrArea), total string length (StrLength), total string width (StrWidth), total string perimeter (StrPerimeter), and total crosslink number (NoXlink). The collagen strings were detected using a connected-component algorithm of binary images from previous image segmentation. Each connected component was fitted with an ellipse, and then the axes of the ellipses were used to divide the strings into long and short as well as thin and thick subgroups. The total lengths, widths, areas, and perimeters of the collagen strings were also measured. We used an image skeleton algorithm to detect branch points for determining the crosslink number.

Group 3: Ratio between the short string and total string numbers (NoShortStr/NoStr), ratio between the long string and total string numbers (NoLongStr/NoStr), ratio between the short string and long string numbers (NoShortStr/NoLongStr), ratio between the thin string and total string numbers (NoThinStr/NoStr), ratio between the thick string and total string numbers (NoThickStr/NoStr), and the ratio between the thin string and thick string numbers (NoThinStr/NoThickStr). We found that these relative ratios improved the system only when absolute (normalized) values were used for Group 2.

### 2.4 Immunohistochemistry staining

Measurement of MMP-9 levels in matched samples was performed by using immunohistochemistry staining and analysis based on established protocols (Wang et al., 2012). For all tissue samples, 5-μm serial paraffin sections were prepared by using the same protocol. First, the sections were immersed in xylol three times for 5 min each to remove paraffin, after which they were hydrated via five consecutive washings with alcohol (100, 100, 95, 80, and 70%, respectively). Second, the sections were immersed in citrate buffer solution (0.01 M, pH 6.0) and heated at 120°C in an autoclave sterilizer for 10 min, naturally cooled for 30 min, then immersed in 3% aqueous hydrogen peroxide for endogenous peroxidase ablation at room temperature for 30 min. The following steps were performed in a moist chamber. The sections were incubated with blocking buffer (Zymed Laboratories Inc., San Diego, CA, USA) containing 20% normal donor bovine serum and 80% phosphate-buffered saline (0.01 M, pH 7.4) at room temperature for 30 min. The bovine serum was then discarded, and the sections were incubated with an optimized dilution of primary antibody anti-MMP-9 (ab38898) at 4°C overnight. The intensity of MMP-9-positive staining in human and animal tissues was quantified in 25 microscopy fields of view (40× magnification), and the means were calculated. Images of all sections were unbiasedly captured under an Olympus BX63 microscope by a single investigator. The area density of MMP-9 positives in the human and animal samples was measured by using an cellSens system (Olympus Co. Ltd., Japan) as previously described (Wang et al., 2012). The results are expressed as MMP-9 area density (area of positives/area of whole field).

## 3 Results

### 3.1 Caseous necrosis and multinuclear giant cells were common features of human tuberculosis granulomas

**Figure 2:**
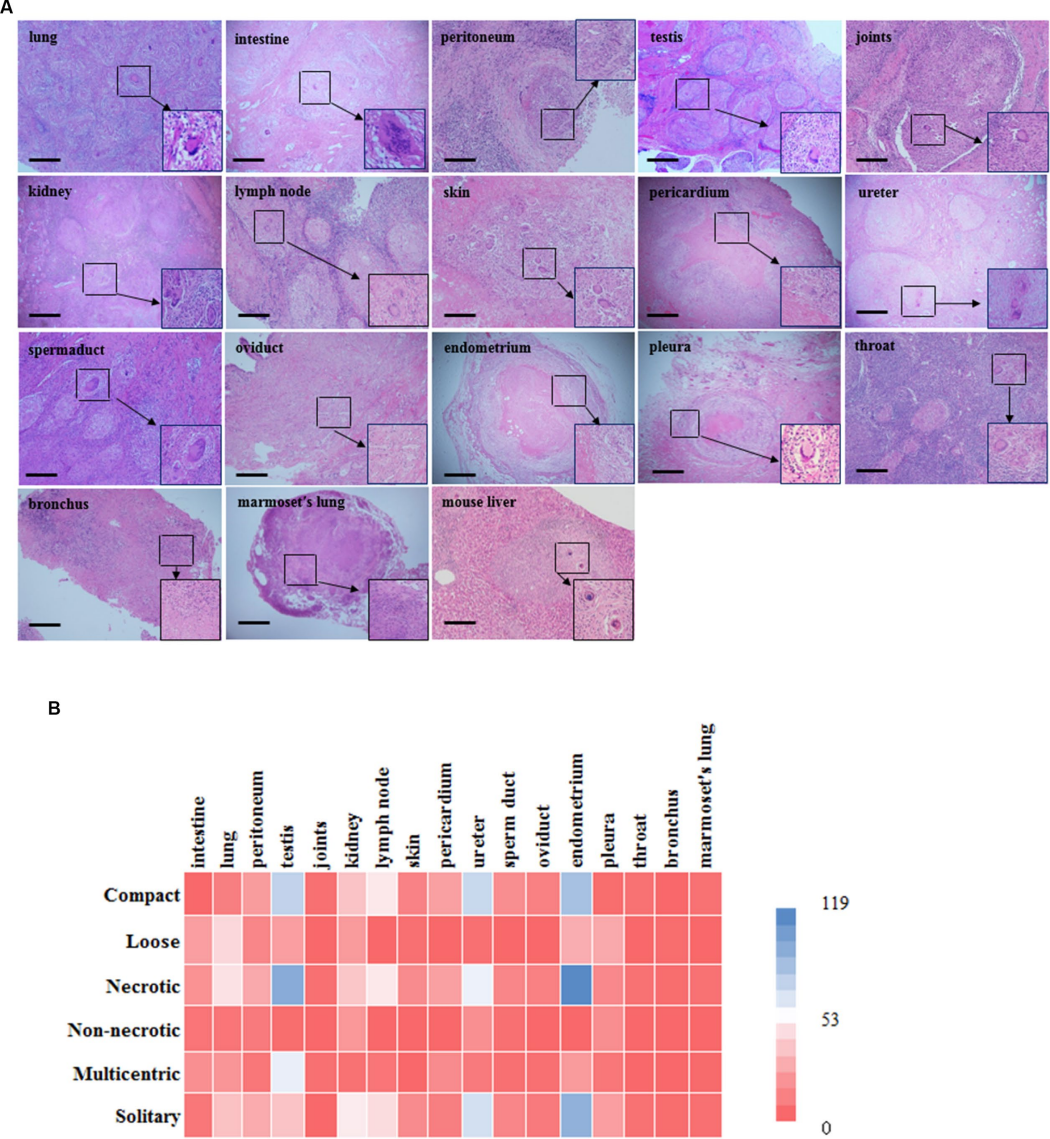
Histopathology examination and characteristics of granulomatous lesions among different organs. **(A)** There is morphology of different granulomas from tissues in human and marmoset by H & E staining. Typical multinuclear giant cells were formed in most human tuberculosis samples excluding the oviduct, endometrium and bronchus (scale bar = 500 μm). However, there were none of characterized multinuclear giant cells formed in *Mtb*-infected marmosets; **(B)** A heat map is shown in the figure. Color bar labelled with number (0 ~ 119) is granuloma counting under microscopy by pathologist. Overall, tuberculosis granulomas are mostly necrotic and solitary granulomas, and structurally they are mostly compact. Among them, necrotic granulomas are mainly seen in the testis and endometrium, while non-necrotic granulomas are mainly seen in the kidney and ureter; The main types of multicentric granulomas include the testis and intestine, while those solitary granulomas include the endometrium and ureter; Granuloma has a relatively compact structure in the testis and endometrium, while loose structures in the lung and pleura. Mouse: C57BL/6 J.

The most common granuloma form observed in our human tuberculosis patient samples was caseous granuloma. Moreover, multinucleated giant cells in tuberculosis granulomas, also known as Langham giant cells, are typical cells in TB, and have a great significance for the development and diagnosis of TB (Silva Miranda et al., 2012; Brooks et al., 2019). For the 16 different tissue types examined, caseous necrosis was detected in all the human tuberculosis samples. Additionally, typical multinuclear giant cells were also observed in most clinical tuberculosis patient samples, excluding the oviduct, endometrium and bronchus samples (Figure 2); however, no multinuclear giant cells were seen in the samples from *Mtb*-infected marmosets (Figure 2). Moreover, the examined granulomas in both human and animal samples exhibited a variety of structures and morphologies, including compact and loose, necrotic and non-necrotic, multicentric and solitary (Figure 2). With Ziehl-Neelsen (ZN) staining, we observed red, rod bacteria distributed in some tuberculosis patient tissues, including the skin, bronchus, and throat. In samples from *Mtb*-infected animals, ZN-positive bacteria were detected in the lungs of marmosets (Supplementary Figure 1). In *S. japonicum*-infected mice, we found diffused necrosis of hepatocytes and egg-encased granulomatous lesions (Figure 2). This result demonstrates the special significance of multinuclear giant cells for clinical human tuberculosis granuloma formation and development, which should be further investigated in future studies.

### 3.2 Masson’s trichrome staining reveals fibrosis surrounding the granulomas

**Figure 3:**
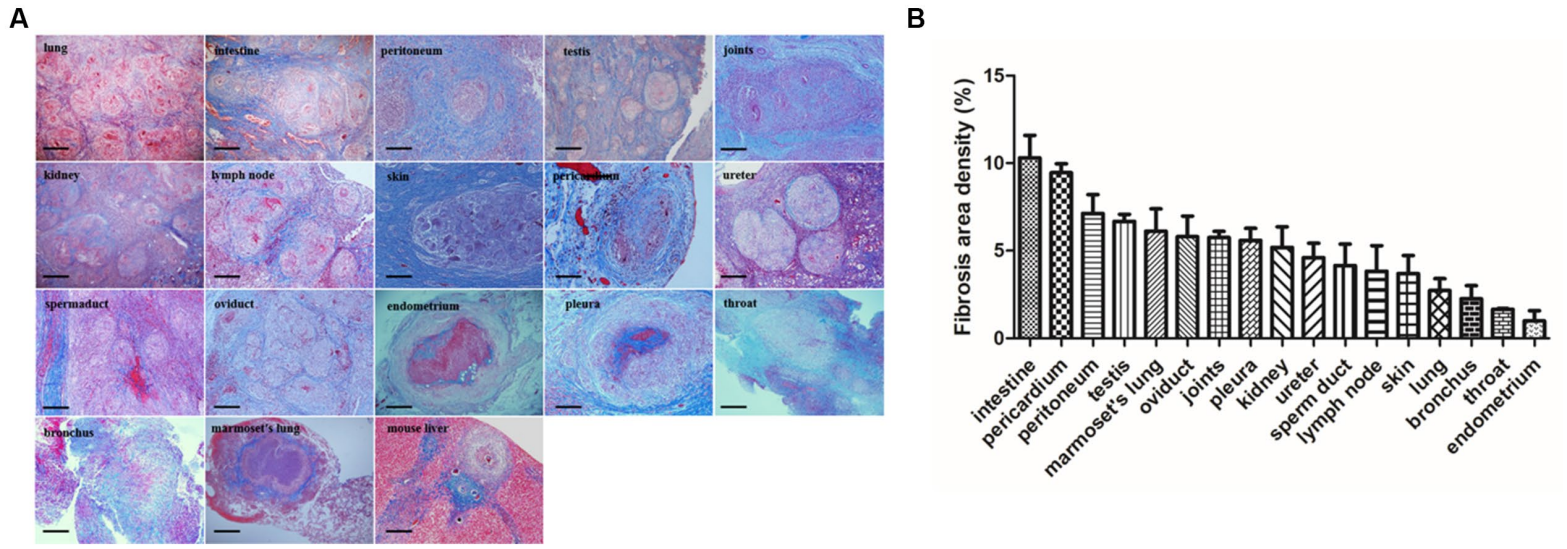
Fibrosis area of granulomas from tissure in clinical TB and MTB-infected marmoset’s lung. **(A)** Masson’s trichrome staining for fibrosis in clinical TB patients and animal tissues. Masson’s trichrome staining revealed fibrosis surrounded the granulomas in most human TB patient tissues as well as in *Mtb*-infected marmoset lungs (scale bar bar = 500 μm). **(B)** The density of fibrosis-positive staining in human and animal tissues was quantified and the means were calculated. The results are expressed as fibrosis area density (area of positives/area of whole field).

Masson’s trichrome staining revealed the typical presence of blue fibers distributed within and surrounding the granulomas in tuberculosis patient tissues, especially in the intestine, lung, peritoneum, testis, joint, kidney, lymph node, skin, pericardium, ureter, sperm duct, oviduct, endometrium, pleura, throat and bronchus (Figure 3A). In *Mtb*-infected animal tissues, Masson’s staining revealed that a fibrotic-ring surrounded the granulomas, with fibers clearly surrounding the granulomas in marmoset tissues (Figure 3A). In *S. japonicum*-infected murine livers, the blue-stained collagen mostly surrounded egg-encased granulomatous lesions (Figure 3A).

### 3.3 SHG image acquisition system

**Figure 4:**
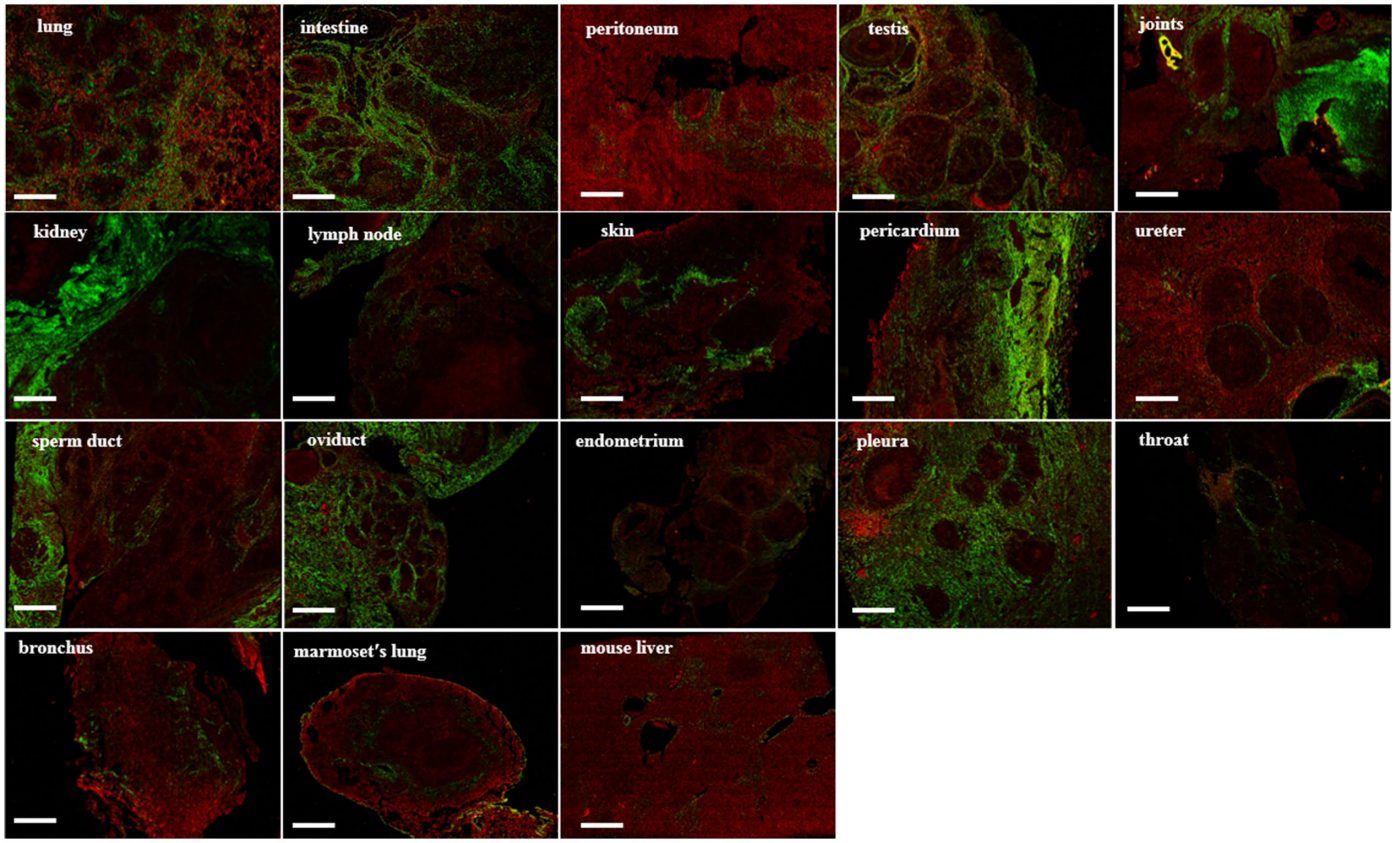
SHG image visualization for fibrosis in tuberculosis human and animal tissues. Green areas represent the collagen under SHG microscope system, while red areas represent the tissue under SHG detection. Scale bar = 500 μm.

**Figure 5:**
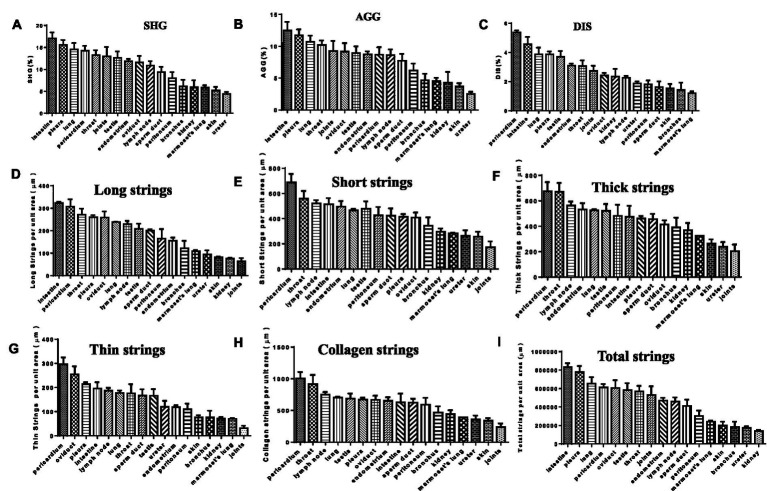
SHG image visualization for fibrosis in TB patients and animal tissues. **(A)** SHG (second harmonic generation): area percentage, indicating the proportion of collagen in the tissue; **(B)** AGG (aggregated Collagen Percentage): area percentage, indicating the proportion of aggregated collagen in the tissue; **(C)** DIS (Distributed Collagen): area percentage, indicating the proportion of aggregated distributed collagen in the tissue; **(D)** Long strings: number of long strings per unit tissue area; **(E)** Short strings: number of short strings per unit tissue area; **(F)** Thick strings: number of thick strings per unit tissue area; **(G)** Thin Strings: number of thin strings per unit tissue area; **(H)** Collagen strings: number of total strings above per unit tissue area; **(I)** Total strings: area of total strings per unit tissue area.

In the SHG image acquisition system, a green region shows the SHG labeling that indicates a fibrotic signal. In multiple tissues of patients with tuberculosis, higher levels of SHG labeling were observed in the intestine, lung, testis, joints, skin, kidney, skin, ureter, oviduct, throat, and pleura (Figure 4). Amazingly, most of the green-stained collagen were distributed directly around the granulomatous lesions (Figure 4). In *Mtb*-infected marmoset lungs, the positive SHG signals were concentrated in the area surrounding the granuloma (Figure 4). The shared parameters of the SHG system, including Agg, Dis, NoShortStr NoLongStr, NoThinStr, NoThickStr, were obtained automatically by the Genesis software system (Figure 5; Supplementary Table 1). Aggregated collagen formed the major component of collagen in each tissue, with the Agg content being higher than that of distributed collagen within each sample (Figure 5). Moreover, the featured forms of collagenous fiber typically presented as short and thick in all examined tissues (Figure 5).

### 3.4 Comparison of fibrosis detection between Masson’s trichrome staining and genesis SHG analysis

**Figure 6:**
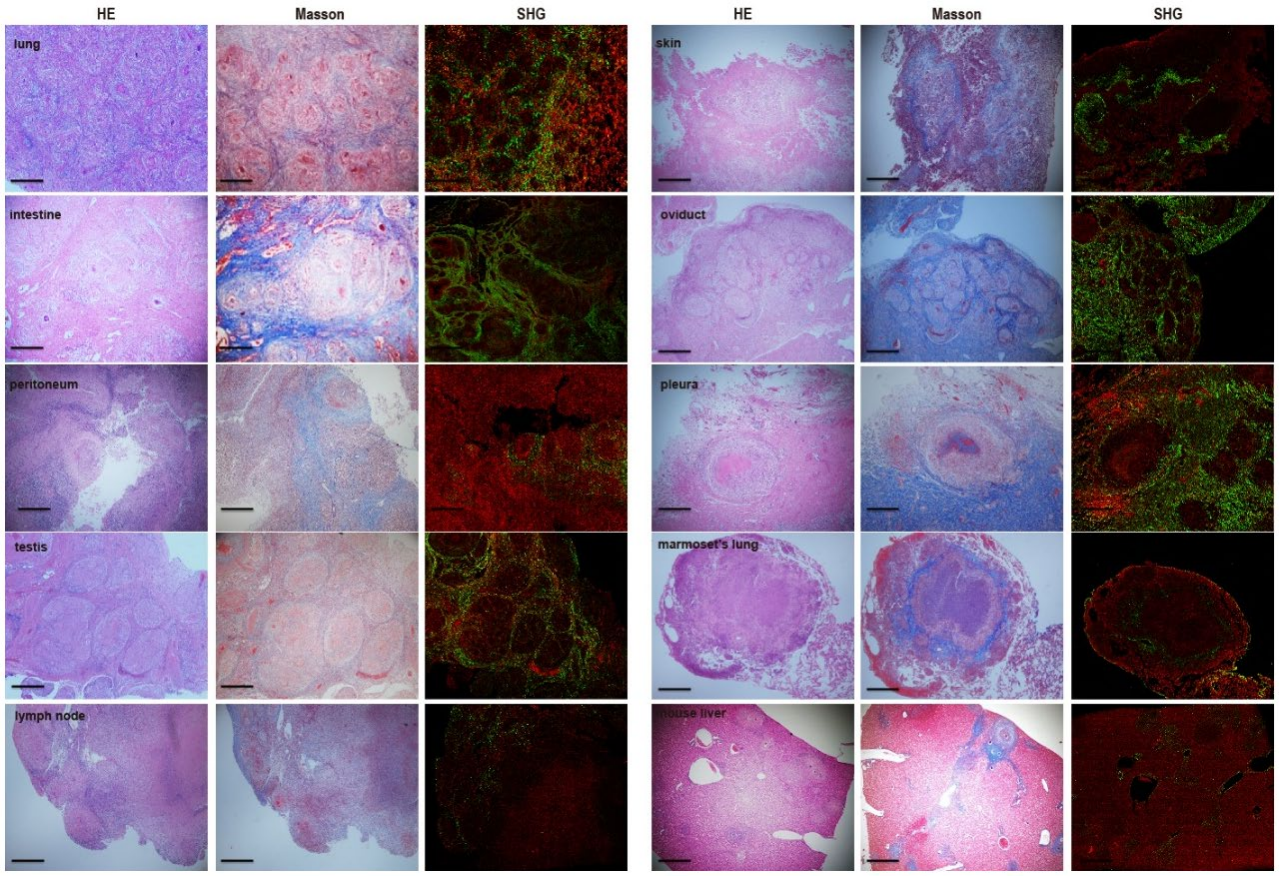
Comparison and consistency of fibrosis between Masson’s trichrome staining and Genesis SHG analysis. Each set of samples are parallel sections of the same tissue. The SHG analysis is in agreement with the distribution of collagens detected by Masson’s trichrome staining. There was a highly fitness for fibrosis detection of those two approaches and SHG analysis displayed powerful capacity for fibrosis examination in TB patients as well as *Mtb*-infected marmosets. Scale bar = 500 μm.

Data from the Genesis SHG automated image acquisition system indicate that the featured collagen was distributed peripherally around the granulomas in human tuberculosis tissues including the intestine, lung, peritoneum, and testis. This finding is not only in agreement with the fibrosis signal distribution detected by Masson’s trichrome staining, but it also matches the known production and distribution of collagens among those tissues (Figure 6). Regarding validation of fibrosis detection in *Mtb*-infected marmoset lungs, the Genesis image analysis demonstrated that collagens directly surround the granuloma (Figure 6). Our results indicate that the SHG image system, an unstained approach, could improve the accuracy of collagen research and be used to describe the fibrosis surrounding granulomas induced by mycobacterial infection.

**Figure 7:**
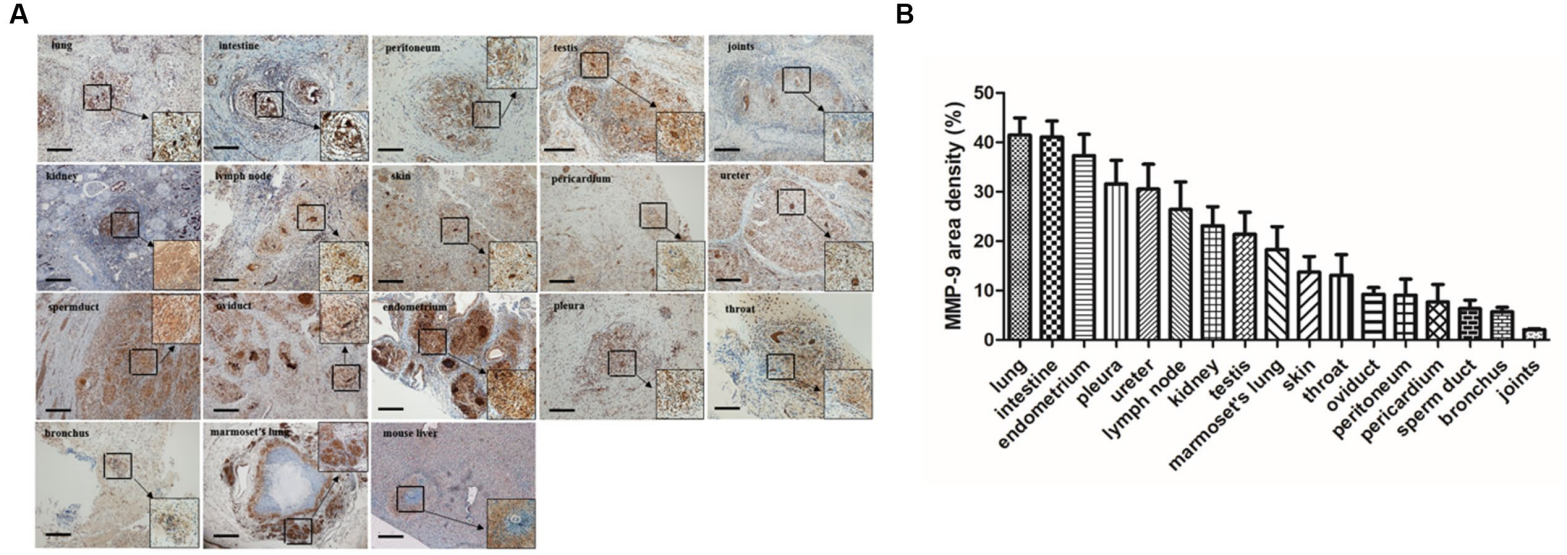
MMP-9 staining and distribution in granulomatous lesions from different tissues. **(A)** Immunohistochemistry staining demonstrated that MMP-9 was extensively distributed in various tuberculosis-associated granulomas including TB patients and marmoset tissues (scale bar = 500 μm). **(B)** The area density of MMP-9 positives in different tissues was quantified and the means were calculated. The results are expressed as MMP-9 area density (area of positives/area of whole field).

### 3.5 MMP-9 expression in various tissue-associated granulomas

MMP-9 is considered to be a critical factor involved in granuloma formation during mycobacterial infection (Ramakrishnan, 2012). Elevated MMP-9 expression is a featured characteristic in idiopathic pulmonary fibrosis, and MMP-9 inhibition was reported to ameliorate pulmonary fibrosis in a humanized immuno-deficient mouse model of idiopathic pulmonary fibrosis (Espindola et al., 2020). In a murine chronic pulmonary granulomatous fibrosis model combined elicited by multiwall carbon nanotube (MWCNT) and mycobacterial ESAT-6 (early secreted antigenic target protein 6), the mice showed exacerbated pulmonary fibrosis and granulomatous pathology and MMP-9 was sharply elevated during this process (Malur et al., 2019). MMP-9-deficiency mice led to reduced peribronchial fibrosis in an allergen-challenged fibrosis model and airway remodeling (Lim et al., 2006). Moreover, MMP-9 activity is actively involved in liver fibrosis (Lachowski et al., 2019) and bleomycin-induced pulmonary fibrosis (Li et al., 2019). In particular, a recent report revealed that MMP-1(-1607G), a member of MMP-9, and its polymorphism could act as a risk factor for fibrosis after pulmonary tuberculosis in Taiwan (Wang et al., 2010). Our results demonstrated that MMP-9 was extensively distributed in various tissue-associated granulomas (Figure 7), implying that MMP-9 was involved in the fibrosis of tuberculosis-associated granulomas including tuberculosis patient’s tissues and marmoset model, which highlights the critical role of MMP-9 in the process of granulomatous fibrosis.

## 4 Discussion

In this study, we gathered samples spanning 16 organ types from a group of patients who presented with clinical tuberculosis and samples of *Mtb*-infected animal lung tissue to study granuloma characteristics and fibrosis-related parameters. We first applied Masson’s trichrome staining to describe the collagen distribution and content. The SHG system was then employed and successfully used to acquire collagen images and quantitative indices of the 16 organ samples from clinical tuberculosis cases. This is the first time to apply SHG microscopy as an unstained and automated system of evaluation and detection of the collagen content and its related parameters in tuberculosis granuloma fibrosis.

When we checked the fibrosis in granulomas by using Masson’s trichrome staining, we observed many collagen proteins distributed around the granulomas in most tissues from human tuberculosis cases. We subsequently confirmed the feasibility of applying the SHG microscopy system to determine the fibrosis characteristics across 16 organ types sampled from patients with tuberculosis and *Mtb*-infected model animals. Finally, we analyzed the consistency between the SHG system and the conventional histopathological technique for studying fibrosis in tuberculosis granulomas and demonstrated that the use of the unstained approach could improve the visualization and digitalization of collagen within granulomatous areas.

In the 16 organ-type samples from human patients with tuberculosis, we found that caseous necrosis was observed overwhelmingly. Multinucleated giant cells, also termed Langham giant cells, are considered as an informative feature for tuberculosis especially have a important role in the TB processes (Silva Miranda et al., 2012; Brooks et al., 2019). In the present study, typical multinuclear giant cells were also observed in most samples, excluding the oviduct, endometrium and bronchus tissue. This finding suggests that multinuclear giant cells could have special significance for the pathological diagnosis of tuberculosis. Previous studies have demonstrated that *Mtb* infection stimulated the production of profibrotic mediators, including TGF-β and collagen, that subsequently mediated granuloma formation (Wang et al., 2011; Vanden Driessche et al., 2013). Additionally, fibrosis has been documented after *Mtb* infection and was shown to be involved in granuloma formation (Dye and Williams, 2010). Moreover, the granulomas from tuberculosis cases examined in the present study exhibited a variety of structures and morphologies, including compact and loose, necrotic and non-necrotic, multi-centric, and solitary. According to the conventional Masson’s staining results, almost all the human tuberculosis samples displayed blue collagen surrounding the granuloma, as did the *Mtb*-infected marmoset lung samples; these findings suggest that fibrosis is involved in the formation of granulomas during tuberculosis development.

To analyze the dynamics and parameters of the collagen involved in granuloma formation, we applied an innovative imaging system that was previously developed and has been widely used as a liver fibrosis assay in animal models and chronic hepatitis patients. SHG and TPEF microscopy, a computer-assisted, fully automated, staining-free method for fibrosis assessment, was employed to assess fibrillar collagen development by using eleven shared parameters for both the early and later stages of liver fibrosis. This method has been previously used to provide quantitative scores incorporating histopathological features of liver fibrosis and facilitate the staging and evaluation of clinical research and management of liver-associated diseases (Denk et al., 1990; Cox et al., 2003; Gorrell et al., 2003; Sun et al., 2008; Tai et al., 2009; Xu et al., 2014; Wang et al., 2015, 2017; Liu et al., 2017). Little is known regarding the fibrosis in tuberculosis granulomas, and data obtained from *Mtb*-infected animals is just beginning to reveal some details. Recently, Warsinske’s group developed a hybrid multi-scale agent-based model to uncover novel critical players and mechanisms driving the formation and development of granuloma-associated fibrosis in *Mtb*-infected animals (Warsinske et al., 2017). Although their findings demonstrate the presence of multiphase mediators in the formation of complicated granuloma structures and morphologies, additional detailed information about fibrosis and its critical parameters still needs to be determined.

With the application of the Genesis program, eleven shared parameters were captured by an automated system, and they revealed that aggregated collagen was the major component in all examined tissue of patients with tuberculosis and *Mtb*-infected marmoset’s lung. Aggregated collagen was reported to surround the granulomatous lesions in severe pulmonary acariasis in a Japanese macaque model (Hiraoka et al., 2001). Although collagen has been reported to play a role in tissue remodeling and granuloma formation during *Mtb* infection (Dye and Williams, 2010; Vanden Driessche et al., 2013), the type of collagen involved in granulomatous lesions was unclear. Our data reveal that aggregated collagen plays an essential role in granuloma formation in both clinical tuberculosis and *Mtb*-infected animals. Furthermore, during granuloma formation, the fasciculus of those aggregated collagens was short and thick in shape.

Several cytokines, such as TNF-α, TGF-β, and MMP-9, are critical for granuloma formation following *Mtb* infection (Iliopoulos et al., 2006; Taylor et al., 2006; Barry et al., 2009; Mootoo et al., 2009; Beham et al., 2011; Fallahi-Sichani et al., 2011; Ramakrishnan, 2012; Roh et al., 2013; Shmarina et al., 2013; Al Shammari et al., 2015; Datta et al., 2015; Ordonez et al., 2016; Warsinske et al., 2017; Walton et al., 2018). Here, we further characterized the profile of those cytokines in clinical tuberculosis human and *Mtb*-infected model animal tissues and found that MMP-9 was highly expressed. MMP-9 is upregulated in the pleural fluid of tuberculous cases and correlated with granulomatous development (Sheen et al., 2009). Furthermore, this cytokine, which is produced by epithelial cells that neighbor *Mtb*-infected macrophages to facilitate macrophage recruitment to the nascent granuloma, also promotes bacterial growth (Volkman et al., 2010). Our data found extensive up-regulation of MMP-9 in various tuberculosis-associated granulomas and demonstrated the significance of MMP-9 in granulomatous fibrosis. Understanding the complicated architecture of all granuloma aspects is critical for developing innovative immunotherapy and medications. Fibrosis aids granuloma formation and contributes to the isolated architecture that is seen in many clinical tuberculosis cases and *Mtb*-infected animal models. Our research reveals that aggregated collagen is the major player involved in granulomatous fibrosis and that most of those aggregated collagens were short and thick in shape.

In summary, the present study first provided insight into the fibrosis dynamics of tuberculosis granulomas by applying an automated quantitative approach, and its results may provide useful insight for developing effective strategies to improve the treatment strategies of tuberculosis and patient outcomes. To the best of our knowledge, although it is the first report that second harmonic generation (SHG)/two-photon excited fluorescence (TPEF) microscopy to determine the fibrosis of tuberculosis-associated granulomas, there are also some limitations in this study. Firstly, due to the influence of anti-tuberculosis treatment, clinical specimen collection time, tissue preservation mode, specimen number and other factors, the results of the study may have certain limitations. Secondly, this study has a preliminary understanding of the fibrotic components of tuberculosis granuloma, but how to use the obtained fibrosis characteristic parameters to provide a basis for the clinical diagnosis and treatment of tuberculosis still needs a lot of clinical research in the future. Finally, due to the complex pathophysiology and functional state of tuberculosis granuloma, the role of various cytokines in the development of tuberculosis is also very complex, and its specific role and mechanism need to be further studied.

## Data availability statement

The raw data supporting the conclusions of this article will be made available by the authors, without undue reservation.

## Ethics statement

The studies involving humans were approved by the Ethical Review Committees of the First People’s Hospital of Yichang. The studies were conducted in accordance with the local legislation and institutional requirements. The human samples used in this study were acquired from the formalin-fixed, paraffin-embedded samples were acquired from the First People’s Hospital of Yichang. Written informed consent for participation was not required from the participants or the participants’ legal guardians/next of kin in accordance with the national legislation and institutional requirements. The animal study was approved by paraffin-embedded lung tissues of *Mtb* aerosol-infected marmosets in this study were acquired from the Tuberculosis Research Section, National Institute of Allergy and Infectious Diseases (Via et al., 2015). The original animal experiment was approved by the NIAID Animal Care and Use Committee approved the experiments described herein under protocol LCID-9 (permit issued to NIH as A-4149-01). The study was conducted in accordance with the local legislation and institutional requirements.

## Author contributions

LS: Data curation, Formal analysis, Methodology, Project administration, Resources, Software, Writing – original draft. DZ: Writing – original draft, Methodology, Data curation, Investigation. HW: Data curation, Formal analysis, Investigation, Project administration, Software, Writing – original draft. XX: Data curation, Formal analysis, Investigation, Visualization, Writing – original draft. WH: Conceptualization, Data curation, Investigation, Methodology, Writing – original draft. JG: Project administration, Resources, Writing – original draft. LV: Conceptualization, Investigation, Project administration, Resources, Writing – original draft. DW: Conceptualization, Formal analysis, Funding acquisition, Investigation, Resources, Supervision, Validation, Visualization, Writing – original draft.

## In Memoriam

This article is dedicated to the memory of our beloved Dr. Weifeng Huang who passed away during the preparation and writing of this manuscript.
