# Supplementary material for: Automated quantitative assay of fibrosis characteristics in tuberculosis granulomas
Source: Front Microbiol. 2024 Jan 3;14:1301141. doi: 10.3389/fmicb.2023.1301141 (PMC10792068; doi:10.3389/fmicb.2023.1301141)
Supplement: Supplementary file 1 [file Table_1.pdf]

Appendix 1|Scales.

Construct and measurement item	Factor loading	CR	AVE
High-performance HR practices			
Your company will follow strict recruitment criteria to attract the right employees	0.694		
Your company will hire employees with long-term development potential	0.650		
Your company attaches great importance to the selection and employment of talents	0.692		
Your company is able to offer a wide range of training programs to its employees	0.774		
Your employees participate in training programs from time to time	0.705		
Your company's training can improve overall employee performance	0.769		
Your company's training is of great help to the improvement of employees' working skills	0.842		
Your company will provide training opportunities and internal promotions to motivate employees	0.716		
Your employees have a clear career path	0.816		
Your company offers a number of open positions for advancement	0.688		
Employees can stay with your company as long as they want	0.661	0.956	0.502
Your company can guarantee almost every employee a stable job	0.802		
Your company has a clear description of the responsibilities of each position	0.768		
Your company often uses objective and quantifiable methods to measure employees' work performance	0.818		
Your company often uses objective, quantifiable results to evaluate employee performance	0.585		
Your company's job evaluation of employees emphasizes long-term performance and team performance	0.535		
The salary of your company's employees is linked to individual (team) performance	0.653		
Your company's employees' bonuses or year-end bonuses are often linked to the company's profits	0.687		
Your company often encourages employee participation in decision making	0.566		
Your company allows employees to make decisions	0.645		
Your company will give every employee the opportunity to propose suggestions for improving their work methods	0.729		
Your company's employees can communicate with their immediate leaders at any time	0.690		
Organizational ambidexterity			
Your company is committed to continuous product upgrading and service innovation	0.795		
Your company is committed to increasing the range of products	0.827		
Your company is committed to continuously exploring new market areas	0.666		
Your company is committed to new technology	0.774	0.932	0.632
Your company is committed to the improvement of existing products and services	0.804		
Your company is committed to improving the applicability of its current products and services	0.845		
Your company is committed to reducing the production costs of current products and services	0.839		
Your company is committed to increasing the production of current products to expand existing markets	0.796		
Organizational Learning			
Management members agree that organizational learning is the key to your company's competitive advantage	0.842		
Improving the learning ability of employees in your company is an investment, not an expense	0.819		

In your company, learning is seen as a key asset necessary to ensure the survival of the organization	0.786		
All employees of your company are committed to achieving organizational goals	0.730		
All employees in your company are actively involved in setting your company's development direction	0.826		
The consensus reached by employees on the organizational vision can guide their strategies in various departments	0.814	0.930	0.573
Your company has common organizational goals	0.606		
Your company's employees dare to question various assumptions in the operation of the organization	0.858		
Your company's employees may question their decisions and long-term activities and engage in innovative thinking	0.600		
Your company attaches great importance to and highly evaluates original viewpoints and opinions	0.625		
Organizational Performance			
Your company has had a relatively high market share in the past three years	0.801		
Your company's profitability has been relatively high in the past three years	0.851		
Your company's productivity level has been relatively high in the past three years	0.905	0.903	0.699
Your company's customer satisfaction has been relatively high in the past three years	0.783		
