# Supplementary material for: Automated quantitative assay of fibrosis characteristics in tuberculosis granulomas
Source: Front Microbiol. 2024 Jan 3;14:1301141. doi: 10.3389/fmicb.2023.1301141 (PMC10792068; doi:10.3389/fmicb.2023.1301141)
Supplement: Supplementary file 2 [file Image_1.pdf]

Supplementary Figure

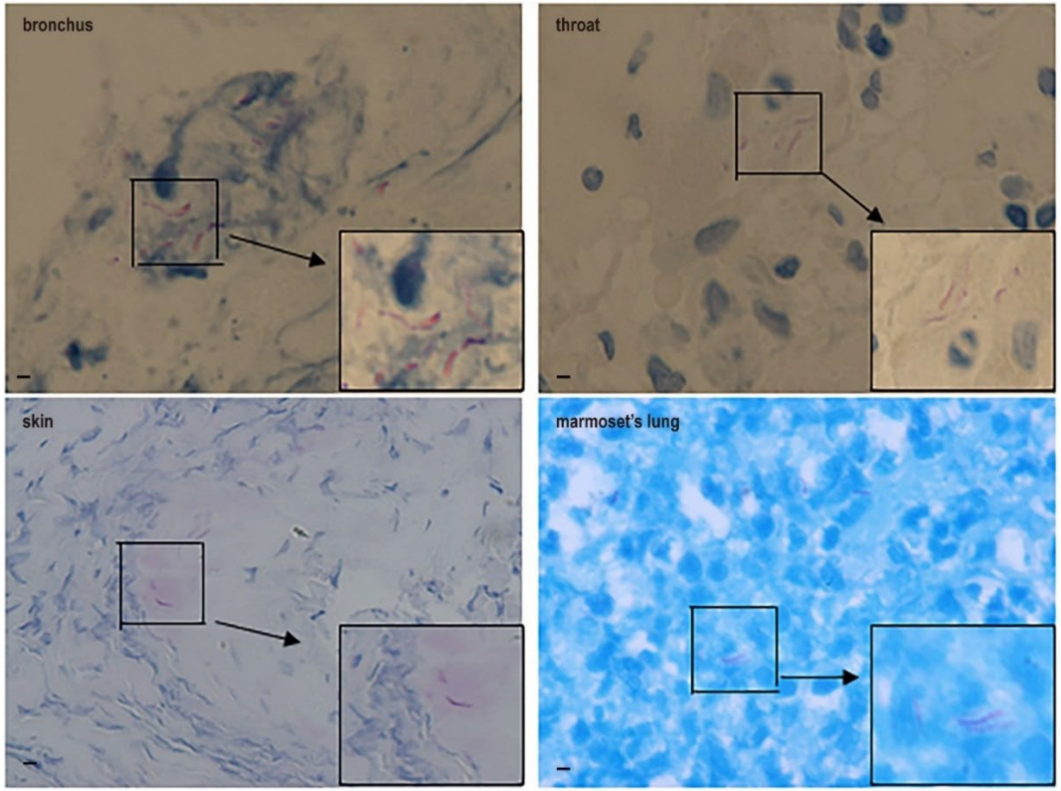

Ziehl-Neelsen (ZN) staining for red-rod bacteria in clinical TB patients and marmoset's lung. scale bar=50μm.
